# Some Points on the Care and Treatment of Mental Defectives

**Published:** 1929

**Authors:** H. L. Ormerod


### The Bristol Medico-Chirurgical Journal

*" Scire est nescire, nisi id me Scire alius sciret."*

SPRING, 1929.

### SOME POINTS ON THE CARE AND TREATMENT OF MENTAL DEFECTIVES.

The Presidential Address, delivered on 10th October, 1928, at the opening of the Fifty-sixth Session of the Bristol Medico-Chirurgical Society.

BY

H. L. Ormerod, M.D., B.Ch., M.R.C.S., L.R.C.P.

My first duty is to thank you for the very high honour you have conferred upon me by electing me President of this Society. It is an honour which I value all the more when I recall the fact that very few general practitioners have held the office.

" Mental defectives, their care and treatment " is a subject that has been somewhat forced upon me during the last few years, and as it is a matter of importance to all members of the medical profession as citizens, I am venturing to use some points under this heading as the basis of my address to you to-night.

*History.* [1]—By the ancients mental defectives were regarded as objects of derision, aversion, or persecution. The appellation "idiot" not only inspired horror and disgust, but meant, for the unfortunate, a forfeiture of all human rights and privileges. So, in the belief that they were accursed of the gods, or in an effort to preserve the integrity of the race, the practice became common, and still exists among certain nations, of allowing the mentally deficient to perish, or, as was done in Sparta, of directly exposing them to a death peril. Traces of this custom, found in the laws of Lycurgus, were not confined to Sparta alone. Cicero intimates its existence among the Romans. At the present day a similar custom exists among certain of the South Sea Islanders, and is common among a tribe of American Indians distinguished for their intelligence, strength, and physical beauty.

The dawn of Christianity was, for the idiot, the first gleam of commiseration and pity, and the charge of St. Paul to "comfort the feeble-minded" is believed by some to apply more to these unfortunates than to those weak in the faith. In direct accord with this, three hundred years later we find the Bishop of Myra, the St. Nicholas of the children of to-day, recognizing and tenderly caring for the idiot and the imbecile. In medieval times, as fools and jesters, they had the freedom of the castles of the great, and wandered unmolested in Europe, as in the Orient.

Again, viewed with superstitious reverence, and even fear, as being mysteriously connected with the unknown, the house into which an imbecile was born was considered blessed of God, and it was a commonly accepted belief that these creatures walked on earth, but held their conversation in heaven. Among the Turks of to-day, and in parts of Ireland and Brittany, the same idea prevails. In Brazil an imbecile in a family is considered more of a joy than a sorrow. Rich and poor alike roam the streets undisturbed, soliciting alms, which are never refused, and in this way, among the poor, an idiot may be the sole support of a family.

Confucius and Zoroaster in their writings both enjoin a tender care of these unfortunates; and the Koran gives this special charge to the faithful: "Give not unto the feeble-minded the means which God hath given thee to keep for them, but maintain them for the same, clothe them, and speak kindly unto them."

During the reign of Edward II we find it enacted that "The King shall have the custody of the lands of natural fools, taking the profits of them, without waste or destruction, and shall find *them* their necessaries, of whose fie soever the lands be holden; and after the death of such idiots, he shall render the same to the right heirs, so that such idiots shall not be aliene, nor their heirs be disinherited, and a portion shall be distributed for his soul, by the advice of the Ordinary." But again persecution follows close upon the steps of superstition, and even in the days of the Reformation we find Martin Luther and Calvin denouncing them as "filled with Satan."

Although many individual acts of kindness may have passed unnoticed during the lapse of ages, the first intimation of organized effort is found in the middle of the seventeenth century, its birthplace France and its cradle the Bicêtre, the present large asylum and hospital of Paris. Obtaining from Anne of Austria permission to use as an asylum for foundlings this ancient château, upon the ruins of which Richelieu had begun the foundation of a military hospital, St. Vincent de Paul and his confrères gathered there the children from the city and the provinces, the homeless, the outcast, and the feeble alike in mind and body.

The first attempt at any mental training was in 1828 by Guggenmoss of Salzburg, but his efforts failed from lack of aid and encouragement. In 1836 Guggenbühl, a young physician of Meilen in Zurich, after working for some time upon cretinism, became convinced that much could be done for mind and body by systematized effort, and determined to found a hospital, which was eventually built on the Abendberg, near Interlaken, in 1842. Accounts of its success quickly spread abroad, and within a decade institutions arose in America, England, Germany, and other countries of Europe.

In England in 1843 articles written by a Dr. William Twining called the attention of his countrymen to the work being accomplished at the Abendberg, and three years later, in 1846, the Misses White opened a private school for defective children in Bath. In 1847 the Rev. Andrew Read, who in the midst of his work for orphans had found time to study also the needs of defectives, journeyed to Abendberg for observation and conference. On his return, at a meeting held on 7th October at which the Lord Mayor of London presided, he was so successful in enlisting the sympathies of many, that a committee was appointed to consider ways and means for practical work. The following April, 1848, Park House, Highgate, was opened as a temporary home, with twenty-seven children. This in 1850 was removed to Essex Hall near

Colchester, and became the asylum for the eastern counties. In 1853 the corner-stone of the present institution of Earlswood was laid, to be followed in the sixties by Starcross for the western counties, the Royal Albert Asylum at Lancaster and the Knowle at Birmingham for the northern and midland counties respectively.

*The Present Day.*—A very definite advance in the care of the mental defective was made on 1st April, 1914, when the Mental Deficiency Act of 1913 came into operation. It[2] was a drastic remodelling of the recommendations of a Royal Commission on the care and control of the feeble-minded, whose report was issued in 1908, after a prolonged investigation. Prior[3] to this Act idiots and imbeciles were provided for by two statutes, namely the Idiots Act 1886 and the Lunacy Act 1890. The education of mentally-defective children was also sanctioned by the Defective and Epileptic Children Act of 1899; but the largest and most important class of all, that of the adult feeble-minded, was not recognized, and the absence of any legalized provision for their systematic care and control caused no little hardship to the defectives themselves, besides being a source of very considerable danger to the welfare of the community.

In 1920 a Mental Deficiency (Amendment) Act was passed, and finally the Mental Deficiency Act 1927. By this Act mental defectiveness is defined as "a condition of arrested or incomplete development of mind, existing before the age of 18 years, whether arising from inherent causes or induced by injury or disease." The mentally defective are divided into the four following groups:—

(1) Idiots.

(2) Imbeciles.

(3) Feeble-minded persons, that is persons in whose case there exists mental defectiveness, which although not amounting to imbecility, is yet so pronounced that they require care, supervision and control for their own protection or for the protection of others ; or, in the case of children, that they appear to be permanently incapable, by reason of such defectiveness, of receiving proper benefit from the instruction in ordinary schools. These are capable in many ways of earning a living under favourable circumstances, but are incapable from mental defect of (*a*) competing on equal terms with their normal fellows, or (*b*) of managing themselves and their affairs with ordinary prudence.

(4) Moral defectives, that is persons in whose case there exists mental defectiveness coupled with strongly vicious or criminal propensities, and who require care, supervision and control, for the protection of others.

In connection with the definitions,[4] the Board of Control desire to emphasize the fact that mental defect, within the meaning of the Act, may exist in persons of some or even considerable intellectual capacity. The criterion, except in the cases of feeble-minded children, is whether the individual is so mentally defective that he or she requires care, supervision and control.

With regard to the moral defectives,[5] this class is one of great practical importance by reason of its comparative frequency and the serious results which may attend its proclivities. A medical practitioner may at any time be brought into contact with a member of this class, either to make a diagnosis, to give evidence in a court of law, or to advise as to certification. At first sight it might appear that the question who were or were not moral defectives within the meaning of the Act would present very little difficulty, but as a matter of fact it is far from simple. Many moral defectives are so cunning, so plausible, and not only so seemingly intelligent but really intelligent, that mental defect, as ordinarily understood, would appear to be quite out of the question. But the definition speaks not of intellectual defect, but of mental defect.

There is a great difference between these terms, for a mental defect is clearly a defect of any of the complex faculties which go to the make-up of mind; whilst an intellectual defect concerns certain particular faculties of mind only. A person who is able to make normal progress at school, who is able to read, write, and reckon, up to the average of his class, who can comprehend and hold intelligent converse upon current events, is commonly regarded as being free from any intellectual defect; he may, nevertheless, have a prominent mental one, for normal mind is made up of more than this. It embraces a higher intellectual quality called by Mercier "wisdom," a quality that guides and governs the higher and more comprehensive phases of conduct, that regulates the main business of life in adaptation to circumstances, that teaches us how to act, so that in the long run, and on balance, our lives will be successful, and in this quality, which is not only a mental but also an intellectual quality, the moral defective is always deficient.

The cardinal feature of moral defectives is the combination of this mental defectiveness, which must have existed before the age of 18 years, with strongly vicious or criminal propensities requiring care, supervision, or control for the protection of others. Broadly speaking, we may say that a criminal act is one which violates the law, whilst a vicious one is one which is inimical to the well-being of society, although not necessarily illegal.

In an interesting paper on this class of defectives Tredgold[6] describes two distinct clinical types: (1) those due to defect of moral sense, and (2) those due to defect of will. Referring to the crimes and vices to which the defect of moral sense may give rise, he says: "To enumerate all of them would practically be equivalent to writing a catalogue of all the misdeeds of which erring human nature is capable; for there are few, if any, which have not been committed by mental defectives of this type." Even as quite young children they are never to be depended on to tell the truth, and although some of their lies are directed towards the achievement or covering-up of some particular wrongdoing, they will lie equally readily about the most trivial things, when the truth would serve their purpose equally well, or even better. Similarly children of 8 or 9 years, or even less, will use the foulest language, they will rob orchards, or gardens, pick pockets, steal from shops, set fire to hayricks, ill-treat and torture animals and children smaller than themselves in the most fiendish manner, take a malevolent delight in annoying those of older years, and generally behave in such a manner as to be a terror to the neighbourhood. Those who go to public schools will be expelled for immoral practices, thieving and incendiarism.

Later, with the growth of knowledge and physical strength, the capacity for crime of these persons becomes increased, and they will embezzle, forge cheques, wreck trains, mutilate cattle, and commit murder. Far from being mentally defective, in the ordinary sense of the word, they are so cunning and scheming in the commission of their crimes as to make detection exceedingly difficult. When taxed with an offence, they will invent the most plausible excuses, and evince a quickness of wit or a degree of imagination of no mean order, whilst in their general attainments some are actually brilliant in certain directions.

There is one quality, however, which never fails to distinguish them, and that is the absence of shame and remorse. Caught red-handed, some of them will admit their guilt in the most bare-faced manner, others will plausibly attempt to explain their actions, and a few may express contrition; but none of them really feel any shame, and they will repeat the offence the next moment, if given the opportunity. Personal honour, social obligation, patriotism, are quite unintelligible to them, and the lack of the restraining influence of moral sense causes the whole of their conduct to be made up of actions based upon the desires of the moment, quite regardless of the effect these may have upon their fellow-beings or upon society.

The second group are those in whom there is a defect of will. Of these we may recognize (*a*) those persons who in consequence of a general weakness of will are unable to resist the ordinary temptations to social misconduct which are inseparable from every-day life, and whose behaviour will be entirely dependent upon the nature of their environment, and (*b*) those persons who are the victims of certain morbid obsessions and imperative ideas, urging them to the commission of certain anti-social acts: included in this class are kleptomaniacs and dipsomaniacs.

The methods of control provided by the Act are two: (1) detention in institutions; (2) placing under guardianship.

Mental deficiency inevitably costs the nation money, for at a low estimate[7] one per thousand of the population need institutional provision, and the kindest and most economical way for suitable cases is institutional care. Neglect to deal with this problem[8] means a cost in misery, vice and industrial inefficiency which cannot be properly estimated; and all the time the problem becomes more serious, and is bound in the long run to affect our position among the great nations of the world.

It is,[9] however, difficult in many cases to convince the members of councils that the expense of maintaining the feeble-minded who cannot maintain themselves must eventually be borne by the community, and that it is a choice between maintenance under improper conditions in Poor Law institutions, prisons, by outdoor relief or an unemployment benefit, or maintenance in institutions where they are under continuous training and care. The present methods of supporting the mental deficients leave them free to cause grave social evils by their delinquencies and degradations, and to produce children who in turn may have to be supported out of the rates. In and out of prison or a Poor Law institution, sometimes receiving outdoor relief, sometimes charity, exposed to temptations they have no power to resist, a misery to themselves and a source of danger to their neighbours, these afflicted persons should be the first charge on any civilized community. Under proper treatment it is possible by early and continuous care and training, not to cure them, but to make them harmless, happy, and to some extent useful.

Defectives[10] are admitted into *institutions* for (1) training, (2) custodial care, according to their grade.

The objects of the treatment are: (1) to arrest their anti-social conduct; (2) to develop their self-respect and ensure their happiness; (3) to teach them various kinds of work for which they are best fitted by reason of their mental and physical condition, and to lead up to their discharge, if possible; (4) to prevent the procreation of children, and so to stop the handing on of the blight to successive generations.

Institutional treatment comes under three heads: (1) medical, (2) occupational, (3) recreational.

(1) *Medical* treatment consists of a regular "overhaul" to treat any disability present—physical drill for men; dancing for women and girls, healthy outdoor exercise.

(2) *Occupation.*—It is astonishing how quickly defectives go downhill mentally and physically if they are not suitably occupied.

(*a*) *Children.*—There is school for those who are able to benefit by continuing their elementary educational work, together with various forms of handicraft, physical training, gardening and organized games. The training of low-grade defectives is not directed to the attainment of a knowledge of reading, writing and arithmetic, nor that of ultimate industrial efficiency; it is nevertheless essentially practical, in that it aims at producing in the children happiness and self-control, and, as a result, makes them no longer a dead weight on those who have the duty of caring for them. To attain this end attention is especially directed to the following points:—

(i.) The inculcation of habits of cleanliness and obedience; (ii.) the fostering of greater self-reliance by teaching the children to help themselves, and not to be entirely dependent on others; (iii.) the provision of legitimate outlets of energy, which if left undirected too often expresses itself in destructiveness and spitefulness; (iv.) the teaching of mutual helpfulness and consideration.

(*b*) *Adults.*—Endeavour to place them in an occupation that will benefit their mental and physical condition, and will turn them from useless and often troublesome members into persons doing their own share of the work of the community. Great importance must be placed on the fact that the amount of work done by any individual is of very much less importance than the fact that he is fully occupied. There is, of course, always domestic work for both men and women, and out-of-door work on the land. It is a great stimulus to defectives actually to achieve the making of something; hence employ as many as possible in workshops at such work as mat-making, brush-making, tailoring, shoe-making, also with the painters and engineers of the institution. Women can work in the laundry, kitchen, bakehouse and administrative quarters, also in the sewing-room and garden.

(3) *Recreation.*—As we have to teach the patients to work, so we have to teach them to play by means of organized games amongst themselves and by matches with outside clubs. The monotony of institutional life can be relieved by concerts, dances, and walks outside.

*Guardianship*, properly provided, seems adequate care and protection for a certain type of case, and makes for the greater happiness of the individual and for economy in the administration of the Act. This form of care is of use for the higher grade defectives, who, whether on licence, in institutions or elsewhere, have shown themselves able to live safely in the community, but who require some measure of support and control. It may also be used for low-grade cases as an alternative to institutional care and supervision, where the home conditions are good and where suitable guardians are available; and may be found useful in those cases where a small regular payment enables parents to provide proper care for the defective at home. Attendance at an "occupation centre" when possible is found to render supervision more effective in guardianship cases.

The principal factors in deciding whether non-institutional care will prove adequate protection are the character of the defective; his past history, temperament, moral characteristics and behaviour; his capacity for work, and the possibility of his procuring suitable work; also the nature of the parent or guardian, the home surroundings and the efficiency of the supervision provided.

There are two other forms of preventive treatment, besides that of segregation, on which I should say a few words, they are (1) sterilization, (2) the regulation of marriage.

*Sterilization*[11] as a means of preventing procreation has been advocated by certain responsible persons, and repudiated by others; but even assuming that this policy were desirable, it is obvious that it could not be put into operation in the absence of a pronounced public opinion in its favour, followed by the necessary legislation.

The advocates of sterilization, and consequent discharge from institutions, as a wholesale remedy for the problem of mental deficiency, and as an alternative measure to segregation, can be answered briefly as follows. (1) The great majority of mentally defective persons require protection, care, and control, on account of their incapacity to maintain an independent existence, apart from the question of the procreation of children. Sterilization will not make a mental defective competent, an inebriate sober, or a criminal moral. (2) Unrestricted freedom in a large number of cases at present under care would lead to a return to the conditions prevalent before the passing of the Mental Deficiency Act. Sterilization would not prevent these persons from leading useless and harmful lives, in and out of Poor Law institutions, prisons, hospitals or refuges, or from spreading venereal diseases. (3) As far as the immediate expense is concerned, the economic burden to the State is not likely to be lessened by discharge of the sterilized mental defective. Care in an institution where his limited capacities are made full use of, and where he may be trained for partial independence, is probably the cheaper method of treatment. (4) Discharge would not increase the happiness of the majority of the patients at present under care in institutions. (5) Sterilization as a compulsory measure is likely to arouse public opposition, but without compulsion it is probable that the objections of the parents of the patients would render the scheme impracticable on any large scale.

An aspect of the question which is perhaps worthy of consideration is whether it is desirable to allow sterilization, not as an alternative, but as a complementary measure to segregation. The procreation of children by defective parents not only causes immediate individual suffering, but is likely to entail a permanent and increasing burden on the community. Children of defective parents, even if not definitely certifiable, are likely both from the point of view of environment and inheritance to find their level in that stratum of society whose increase at the expense of the self-supporting sections constitutes a serious menace to civilization and progress. That this danger cannot at present be entirely eliminated, except by segregation in colonies, has been proved.

On the other hand, it is clearly impossible under the present law, and the state of public opinion, to provide institutional care for all our defective population, even if it were desirable, and the question arises whether in cases where permanent institutional care is now only necessary in order to prevent the risk of procreation, sterilization, followed by return to some form of community life, on licence, under supervision or guardianship, might not be the most humane and expedient course to adopt.

Taking[12] all things into consideration, it has been estimated, on high authority, that the number of defectives now detained in institutions who could be discharged merely by reason of sterilization cannot be higher than 3 to 5 per cent., and probably the smaller figure is the more correct of the two.

To sum up:[13] whether voluntary sterilization would be accepted by defectives and their parents, whether it would be sanctioned by public opinion and Parliament, whether it would increase the health and happiness of those sterilized, are questions which call for careful study and investigation. The aspect of the question which is more frequently emphasized is the economical one. To this undue weight is being given, for sterilization would not obviate the need for supervision and industrial training, and the number for whom it would render institutional provision unnecessary is greatly exaggerated.

There can be no two opinions[14] as to the undesirability of the *marriage of defectives*, who have been certified as unable to manage themselves and their affairs, or as needing care, supervision, and control. To place such persons in control of others, especially of helpless children, is the height of unwisdom, and the negation of the aims of a civilized community. It has been suggested that it should be made a punishable offence to marry or connive at the marriage of any person known to be certified as a mental defective, but even if the law were so altered, it is very doubtful if public opinion is sufficiently informed to enforce it.

It may be said that legal prohibition would only partially check their propagation, because a large number of their offspring are illegitimate; but that considerable benefit to the State would result cannot be doubted. The procreation of children by unmarried defectives, deplorable as it is, has not, as a rule, the same evil consequences to the children as those of married defectives. In the former case the children are generally brought up apart from their parents under Poor Law or other institutional care, and at the present day are given the chance of as full development as possible, morally, physically and mentally. On the other hand, those who are the offspring of married defectives remain under the control of persons who are incapable of taking care of them; and they are consequently exposed to the hardships, neglect or ill-treatment that the mental condition of their parents renders inevitable.

Laws[15] regulating the marriage of mental defectives do already exist in some European countries and in several of the states of America, and although it is difficult to obtain definite information as to their working, and opinions differ somewhat as to their value, there seems to be little doubt that on the whole they have been productive of good, and have not only helped to educate the conscience of the community, but have prevented many undesirable persons from getting married.

I do not think that I can do better than close with the following passage from Tredgold's *Mental Deficiency.*[16] He says:—

"I am convinced that the nation which wishes to escape degeneracy will sooner or later have to give serious attention to the matter of the innate constitution of its citizens, and the manner in which that innate condition may be controlled by laws concerning marriage.

"So long as we are content to raise no voice against the marriage of the diseased, the degenerate, the habitual criminal, and the chronic pauper, and are willing to educate, feed, clothe, and ultimately pension as many offspring as these persons see fit to produce; so long as legislation is permitted a free hand in doing everything calculated to diminish parental and social responsibility, and to strike at the very root of any incentive to labour; so long as our lawmakers and would-be philanthropists are blind to the folly of transferring the burdens and penalties inevitably following carelessness, improvidence, indifference, drunkenness and unlimited selfishness from the shoulders of those upon whom they should rightly fall to the careful, provident and industrious members of the State—then so long will these classes (and these qualities) continue to be perpetuated, and their numerical ascendancy is simply a question of time."

### REFERENCES.

[1] Barr, *Mental Defectives.*
[2] Leach, *Mental Deficiency Act*, 1913.
[3] Tredgold, *Mental Deficiency*, 4th Ed., 1922, p. 491.
[4] Board of Control, Circular No. 702.
[5] Tredgold, *The Practitioner*, xcix., 1927, p. 43.
[6] *Ibid.*, pp. 45, 48.
[7] Board of Control, 13th Report.
[8] Dr. McCutchen, *Journal of Mental Science*, 1925, lxxi., p. 703.
[9] Board of Control, 13th Report.
[10] Dr. McCutchen, *Journal of Mental Science*, 1925, lxxi., p. 694.
[11] Board of Control, 12th Report.
[12] Dr. F. D. Turner, Board of Control, 13th Report.
[13] Board of Control, 13th Report.
[14] *Ibid.*
[15] Tredgold, *Mental Deficiency*, 4th Ed., 1922, p. 537.
[16] *Ibid.*, pp. 538, 539.

